# Molecular Epidemiology of *Streptococcus pneumoniae* Serotype 1: A Systematic Review of Circulating Clones and Clonal Clusters

**DOI:** 10.3390/ijms26052266

**Published:** 2025-03-04

**Authors:** Onyansaniba K. Ntim, Eric S. Donkor

**Affiliations:** Department of Medical Microbiology, University of Ghana Medical School, Korle Bu, Accra P.O. Box KB 4236, Ghana

**Keywords:** *Streptococcus pneumoniae*, serotype 1, sequence type, clonal complex, invasive pneumococcal disease

## Abstract

*Streptococcus pneumoniae* serotype 1 is one of the most prevalent serotypes commonly associated with invasive pneumococcal disease cases and outbreaks worldwide. Several sequence types of this serotype have been identified globally, including those exhibiting both high virulence potential and antimicrobial resistance profiles. This systematic review presents the global distribution of clones of pneumococcal serotype 1, describing their circulating patterns in various regions in the world. A database search was conducted in Google Scholar, PubMed, Scopus, ScienceDirect, and Web of Science using keywords related to *Streptococcus pneumoniae* serotype 1. The inclusion criteria entailed peer-reviewed studies published in English describing the utilization of at least one molecular genotyping tool to identify *S. pneumoniae* serotype 1 clones based on their sequence types. Data extracted were managed and analyzed using Microsoft Excel 365 (Version 2108). Forty-three studies were finally included in the systematic review. A total of 103 MLST serotype 1 sequence types were identified in 48 countries. These clones were widely reported to be associated with invasive pneumococcal diseases. Globally, ST217 and ST306 clonal complexes (CC217 and CC306) were the predominant lineages of serotype 1 sequence types, exhibiting distinct continental distribution patterns. CC217, characterized by ST217, ST303, ST612, ST618, and ST3081, was predominant in Africa and Asia. ST306 clonal complex, which is grouped into ST306, ST304, and ST227 were mostly found in Europe, Oceania, North America, and some countries in South America. ST615 was predominant in Chile, Peru, and Argentina. The hypervirulence nature of serotype 1, coupled with its complex genetic diversity, poses a significant public health threat. Our findings emphasize the need for enhanced surveillance and targeted interventions to mitigate the spread of these hypervirulent clones, ultimately informing evidence-based strategies for disease prevention and control.

## 1. Introduction

*Streptococcus pneumoniae*, also known as pneumococcus, is a Gram-positive bacterium that colonizes the nasopharynx as part of the commensal flora of the upper respiratory tract in humans [1,2]. Nasopharyngeal colonization is asymptomatic [3,4], but plays an important role in the horizontal transmission of pneumococcus via close contact [2]. Young children [5,6], smokers [5,7,8,9], asthmatics [5,7], and those with acute upper respiratory tract infections [5,6,7], are particularly at risk of pneumococcal colonization. Children are the major carriers of pneumococcus [10,11]. *S. pneumoniae* is highly capable of evading the host’s immune response and spreading from the site of colonization to other sterile sites to cause invasive pneumococcal disease (IPD) [1,2,5]. Pneumococcal carriage is a major risk factor for the development of IPD [1,2,12]. As a leading cause of invasive diseases, *S. pneumoniae* is responsible for significant morbidity and mortality manifesting as meningitis, pneumonia, bacteremia, and sepsis [13,14]. Risk groups, including young children (6 years old), elderly people, and immunocompromized patients (e.g., individuals with HIV or cancer), are mostly affected by IPD [5,15,16,17]. Since pneumococcal colonization is highest in children, they tend to+ face the highest burden of these diseases. According to the World Health Organization (WHO), IPD-associated deaths in children < 5 years old account for approximately one million out of 1.6 million cases annually, with the majority of these casualties occurring in low- and middle-income countries [16,18].

Currently, one hundred different serotypes of *S. pneumoniae* have been characterized based on the differences in the structure of pneumococcal capsular polysaccharides [19,20]. However, only a few of these serotypes cause the most invasive pneumococcal infections (IPD) [21,22]. Among them is pneumococcal serotype 1, one of the first discovered serotypes in the 20th century [19,23,24]. It is rarely recovered from healthy individuals but is highly prevalent in IPD cases reported in various regions worldwide [23,25]. The low serotype 1 colonization rate in healthy people suggests a rapid progression to invasive disease upon carriage [25]. Pneumococcal serotype 1 mainly causes pneumonia, bacteremia, and meningitis but has also been found to be associated with empyema [26,27,28,29]. This level of invasiveness poses a major risk to public health. Molecular genotyping techniques, such as multilocus sequence typing (MLST) [19,30,31], pulsed-field gel electrophoresis (PFGE) [31,32], and a multilocus variable number of tandem repeat analysis (MLVA) [33,34], have revealed the complex genetic diversity within *S. pneumoniae* population. Several studies utilizing MLST tools have identified specific sequence types (STs) of serotype 1 associated with IPD cases globally, with varying geographical distributions [23,35,36]. Furthermore, serotype 1 IPD incidence may increase within countries as a result of clonal spread facilitated by these genetic variations. Despite the growing research on *S. pneumoniae* serotype 1, a comprehensive analysis of its clonal distribution is lacking. Knowledge of the clonal distribution of serotype 1 in the various continents will inform evidence-based strategies for pneumococcal disease surveillance and targeted vaccine development. To fill this gap, we consolidated currently available data globally to describe the circulation of serotype 1 clones and their clonal complexes.

## 2. Results

### 2.1. Search Results and Selection Process

**Figure 1:**
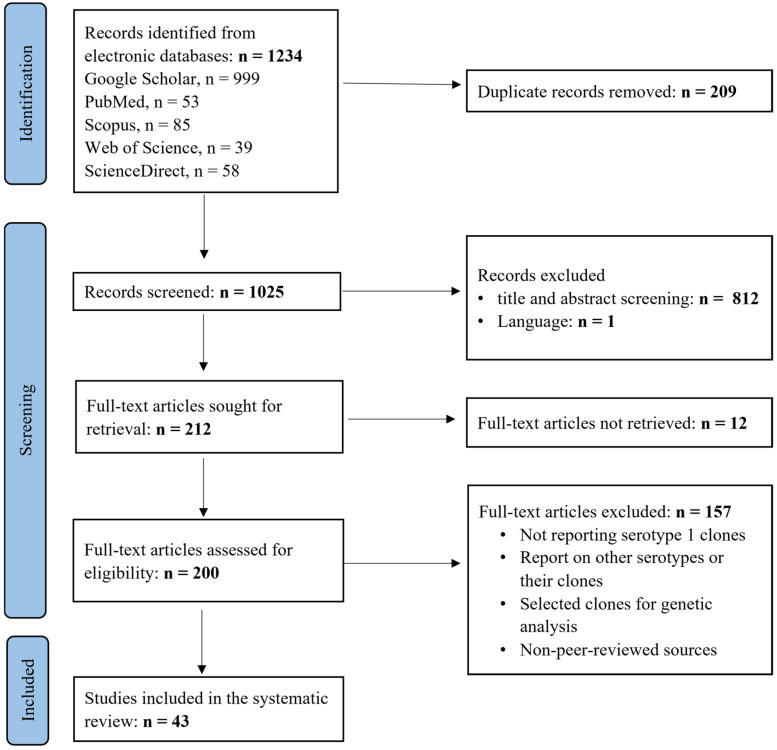
PRISMA flow diagram of the study selection process.

A total of 1234 records were identified from all the five electronic databases (Google Scholar, PubMed, Scopus, Web of Science, and Science Direct) that were searched. Two hundred and nine (209) duplicate records were removed, leaving 1025 unique records to be screened. Of the 1025 unique records, 813 were excluded based on their title, abstract, and language, and the remaining 212 records were sought for full-text retrieval. After full-text retrieval, 157 articles were excluded following the full-text eligibility assessment. Finally, 43 studies were included in the systematic review (Figure 1).

### 2.2. Description of Included Studies

Most of the data analyzed in this study were obtained from single-center studies conducted mainly in 19 countries (n = 38 studies). The majority of the studies were found in the United Kingdom (n = 5), Spain (n = 5), the United States (n = 5), and Gambia (n = 5). Five articles were multicenter studies, including two intra-continental surveys that grouped data from countries in sub-Saharan Africa and West Africa, respectively, one intra-continental survey that described data from the individual countries, and two inter-continental multicenter studies that also reported data from the individual countries. Of the 43 studies, 19 studies (44.2%) focused on participants of all ages (children to adults), 16 studies (37.2) on children only, and two studies (4.7%) on adults only. Six studies did not specify the age group of the included participants. Most studies (n = 29, 63%) described IPD among participants, while four described pneumococcal carriage. Ten studies reported on two or more isolate types (IPD and NIPD, n = 6; IPD and carriage, n = 3; IPD, NIPD and carriage n = 1). The PCVs evaluated includes PCV7, PCV9, PCV10, PCV13, PPV23, and PPV24. Studies employed polymerase chain reaction (PCR), whole genome sequencing (WGS), or a combination thereof, alongside multilocus sequence typing (MLST) analysis for *S. pneumoniae* to determine their sequence types. Table 1 summarizes the characteristics of the included studies.

### 2.3. Pneumococcal Serotype 1 Clones Reported Worldwide

**Figure 2:**
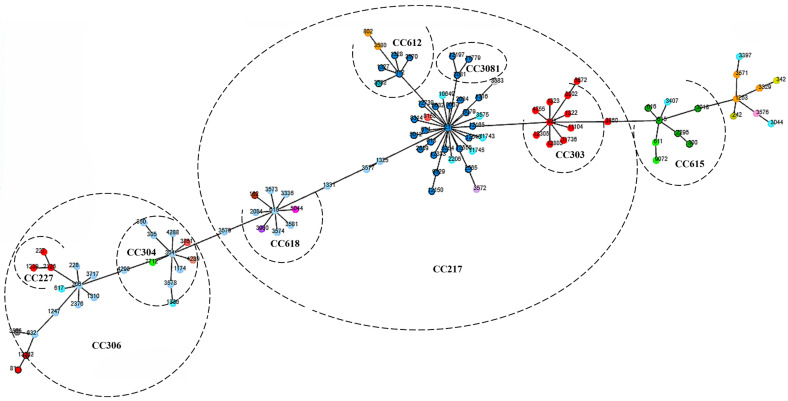
Minimum spanning tree describing the relationships between the various clones identified in this review.

Table 2 summarizes the geographical distribution of studies and sequence types (ST) identified in the various continents. The BURST analysis identified three main clonal complexes (CCs): CC217, CC306, and CC615 (Figure 2). The molecular characteristics of 103 pneumococcal serotype 1 clones identified in this study have been described in the Supplementary Materials (Table S1).

#### 2.3.1. Africa

Our search identified seventeen (17) studies describing pneumococcal serotype 1 STs from African countries (Table 2). BURST analysis revealed that clonal complex 217 (CC217) is the largest group, comprising five main clusters: CC217-ST217, ST303, ST612, ST618, and ST3081. This group was predominantly reported in Africa. The ancestral clone, ST217, was predominantly associated with pneumococcal meningitis in Niger [66], Ghana [49,50], Burkina Faso [64], and Egypt [74]. Additionally, ST217 was widely responsible for several IPD cases described in South Africa [44,54], Kenya [44,55], Ethiopia [35], Mozambique [74], Malawi [70], and Gambia [42,63,74]. Carriage of serotype 1 ST217 was reported in Gambia [42], Kenya [55], Ethiopia [74], and Nigeria [74]. Single locus variants (SLV) of ST217, such as ST613 and ST614, were identified in Kenya as associated with IPD cases and carriage to a lesser extent [44,55]. Other SLVs including ST1316, ST1325, ST2034, ST2206, ST2839, ST3575, ST5012, ST5632, ST8158, ST8314, ST9067, and ST9529, as well as double locus variants (DLVs) like ST12150 and ST12739 were identified in countries in sub-Saharan Africa [37,42,50,54,74].

**Figure 3:**
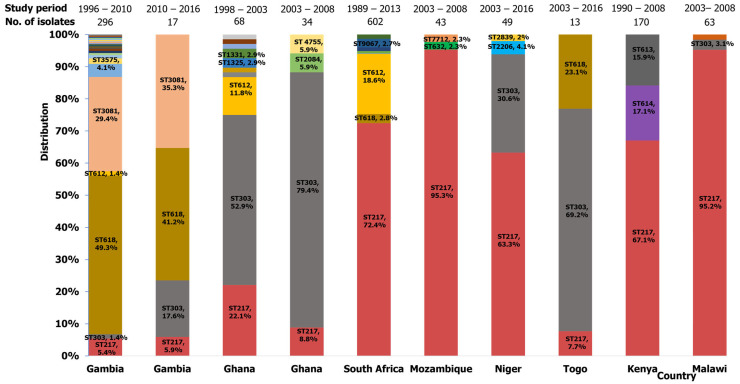
Distribution of serotype 1 isolates identified in Africa.

**Figure 4:**
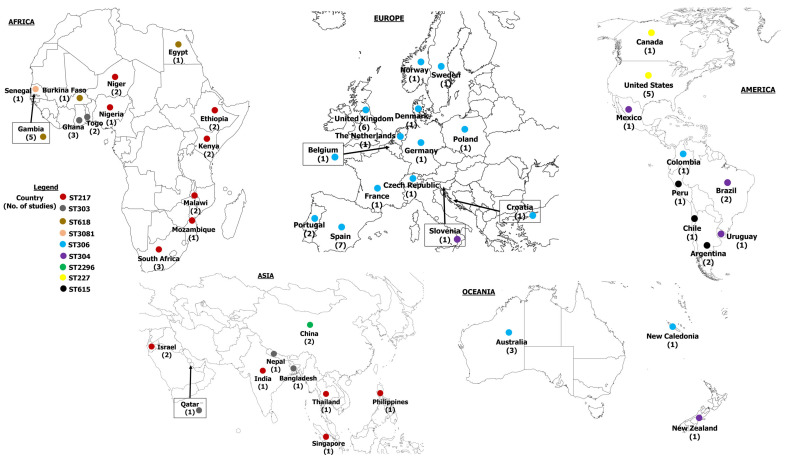
The global circulation of dominant clones of *S. pneumoniae* serotype 1.

ST303, a triple locus variant (TLV) of 217, was recovered from IPD cases in Gambia [52,71,74], Ghana [49,50,74], Malawi [74], Niger [74], and Togo [72]. Its SLVs, ST1322, ST1323, and ST4755 were reported in Ghana [50,74] while others including ST11104, ST11736, ST12305, and ST12803 were identified in sub-Saharan African countries [37]. ST3081 (TLV of 217) and its SLVs, ST11779, and ST12197 were identified in Gambia [42,52,74], Senegal [74], and other countries in sub-Saharan Africa [37]. ST612 (TLV of 217) was described in studies from Ghana [50], Gambia [42], and South Africa [44,54,74], while its SLVs, including ST1327, ST1328, and ST3570, were found in Ghana [50] and Gambia [42]. Another TLV of 217, ST618, was reported in Burkina Faso [64], Egypt [74], Gambia [42,52,63,71,74], South Africa [54], and Togo [72]. SLV of ST618, such as ST1331, was only reported in Ghana [50], ST2084 in Ghana and Gambia [42,74], and ST3336, ST3573, ST3574, ST3577, and ST3581 in Gambia [42]. Some studies from South Africa reported ST304, ST306, and ST611 [44,54]. The distribution of clones reported in this region has been summarized in Figure 3. CC217 clones dominant in this region were ST217, ST303, ST618, and ST3081 (Figure 4).

#### 2.3.2. Europe

**Figure 5:**
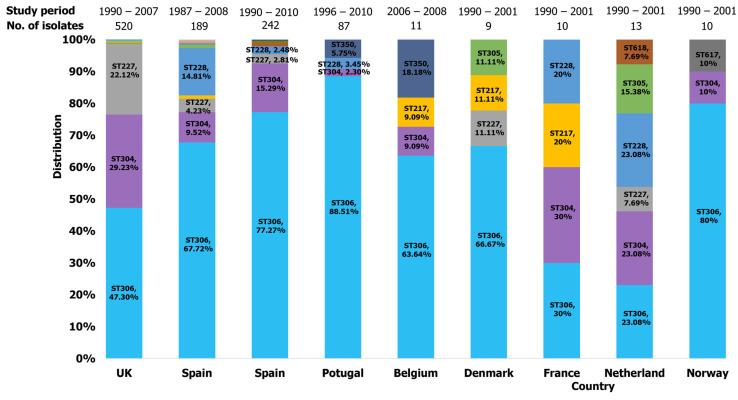
Distribution of serotype 1 isolates identified in Europe.

A total of 16 studies from the European region were identified (Table 2). Clonal complex 306 was the second largest group based on the BURST analysis. It was divided into three main clusters: CC306-ST306, ST304, and ST227. This group was widespread and diverse across Europe, with a significant presence in cases of IPD reported in the 16 studies. The ancestral clone, ST306 was the predominant clone in the region and frequently associated with IPD cases in the United Kingdom (UK) [22,59,60,69], Spain [28,40,41,58,61], Sweden [74], Portugal [46,73], Poland [44], Norway [44], Netherland [44], Germany [44], France [44], Czech Republic [44,57], and Croatia [74]. Alongside ST306, its double locus variant (DLV), ST304 was notably reported in France [44], Netherlands [44], Norway [44], Portugal [73], Slovenia [74], Spain [28,40,41,58,61,74], and the UK [22,59,60,69]. ST227 was reported in Denmark [44] and the UK [22,44,60,67,69], while ST228 was described in Portugal [73], France [44], and Croatia [74]. Both ST227 and ST228 were identified in Spain [40,41,58,61,74] and the Netherlands [44]. Other STs with CC306, including ST1247 (DLV of 306), ST1310 (SLV of 306), and ST2376 (SLV of 306), were reported in the UK [22] and Spain [40,61]. Additionally, ST617 (SLV of 306), ST1239 (SLV of 227), and ST3861 (SLV of 304) were reported in Norway [44], UK [22] and Spain [41], respectively. Clonal complex 217 (CC217) was also identified in European countries, with ST217 widely documented in studies reporting IPD cases in the UK [22], Denmark [44], France [44], Germany [44], and Spain [40,41]. ST618 was only reported in the Netherlands [44] but was identified along with ST614 (SLV of 217) in Spain [58]. To a lesser extent, ST300 (DLV), ST616 (SLV), and ST3860 (DLV) of ST615 were documented in reports from the UK [44,69], Poland [44], and Spain [41], respectively. Regarding serotype 1 carriage, ST306, ST304, ST350, ST228, and ST217 were identified in nasopharyngeal samples from healthy children attending nursery schools in Belgium [45] and Portugal [39]. Figure 5 represents the proportions of clones identified in European countries. ST306 was dominant in almost all countries in Europe except Slovenia, where ST304 was found to be dominant (Figure 4 and Figure 5).

#### 2.3.3. Asia

**Figure 6:**
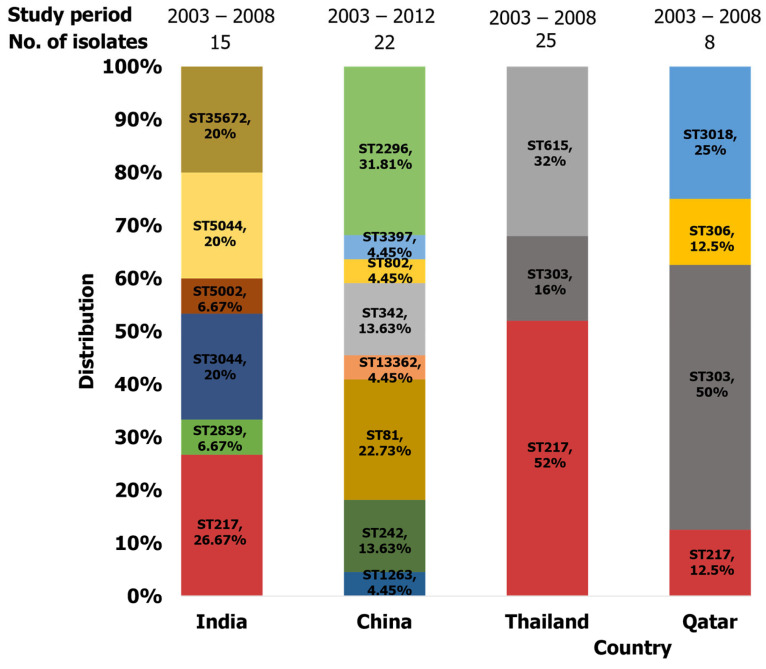
Distribution of serotype 1 isolates identified in Asia.

Pneumococcal serotype 1 clones reported in five studies conducted in Asia revealed a predominant clustering of member clones of CC217 (Table 2). This group encompasses subgroups such as CC303, CC618, and CC3081. In India, clones belonging to this group, including ST217 and its variants ST2839 and ST5002, were identified along with ST5044 related to ST618 and ST5672 (SLV of 303) [74]. Following the introduction of PCV7 in Singapore, serotype 1 strains causing meningitis and sepsis were primarily ST217, ST303, ST5044, and ST5254 [48]. In other Asian countries, such as the Philippines, Bangladesh, Nepal, and Thailand, STs belonging to CC217, including ST217, ST303, and ST3081, were detected [74]. Notably, ST615, part of the CC615 group, was also identified in clinical isolates in Thailand [74]. In Qatar, clinical isolates from blood specimens were represented by ST217, ST303, and ST3081, as well as ST306 (CC306) [74] (Figure 5). Porat et al. found that serotype 1 ST217 was the predominant clone in the southern region of Israel, accounting for cases of acute otitis media (AOM) and IPD in children under five years old [68]. Notably, ST304 was also identified in children with IPD in that study. In China, ST2296 was predominant along with singletons such as ST81, ST242, ST342, ST1263, ST3397, and ST13362 identified in isolates from invasive and non-invasive pneumococcal disease [36]. ST217 and ST303 were the dominant clones reported in Asia (Figure 4 and Figure 6).

#### 2.3.4. Americas

**Figure 7:**
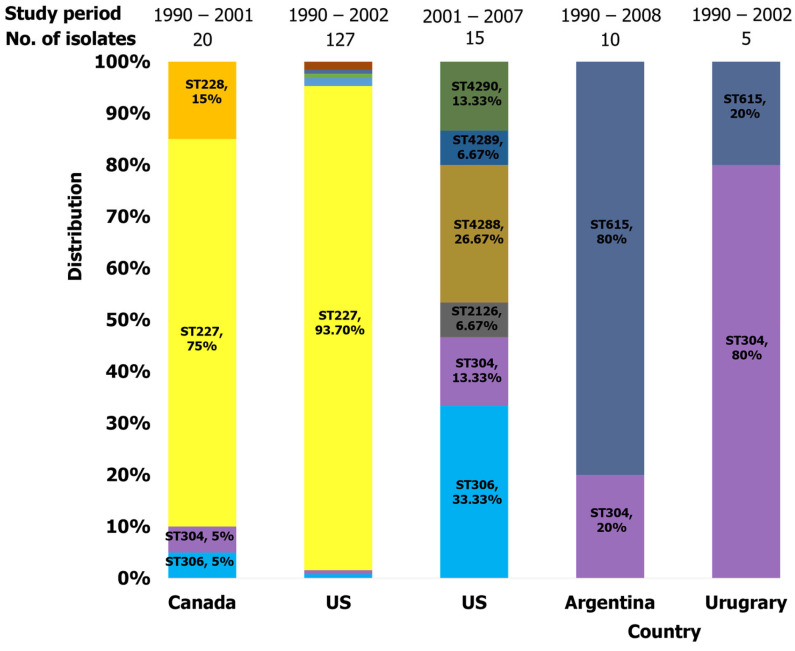
Distribution of serotype 1 isolates identified in the Americas.

Six studies reported pneumococcal serotype 1 clones circulating in North American countries (Table 2). Related clones of clonal complex 306 were widely reported in this region. Studies conducted in the United States from 1990 to 2002 consistently reported ST227 (DLV of 306) as the dominant ST (Figure 7). For instance, a study assessing the clonal compositions of invasive pneumococcal serotypes in the United States in 1999, 2001, and 2002 found that ST227 was the prevalent serotype 1 isolates [62]. This was consistent with the findings of Brueggemann and Spratt [44] and Gonzalez et al. [43], confirming ST227 as the most prevalent serotype 1 clone associated with cases of IPD. However, from 2003 onwards, multiple STs closely related to ST227, including ST304, 306, 2126, 4290 (SLV 306), 4288, and 4289, were reported in cases of Pediatric Pneumococcal Empyema (PPE) in Utah [27] (Figure 7). Like in the United States, ST227 was the predominant ST in Canada [44]. In contrast, in Mexico, cases of serotype 1 IPD were predominantly associated with ST304 [57].

Three studies reported pneumococcal serotype 1 clones in six countries from South America (Table 2). These studies documented the widespread of member clones of CC306 and CC615. In Argentina and Uruguay [57,74], the dominant clones identified in isolates of IPD cases were ST615 and ST304, respectively. ST615 clone was reported in isolates from blood and pleural fluid in Chile [44] and Peru [74]. According to Zemlicková et al. [57], serotype 1 IPD in Brazil and Colombia was caused by ST304 and ST306, respectively. The percentages of STs circulating in the American region are illustrated in Figure 7. We found ST227, ST304, ST306, and ST615 to be dominant in countries in this region (Figure 4).

#### 2.3.5. Oceania

**Figure 8:**
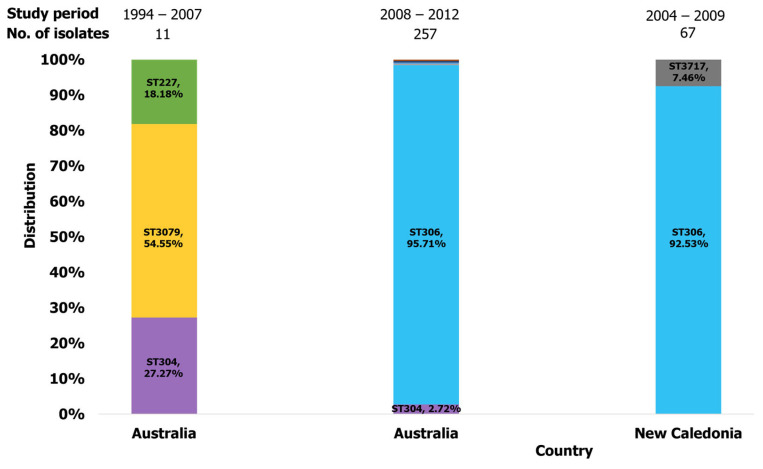
Distribution of serotype 1 isolates identified in Oceania.

Our search identified six studies from Oceania that reported clones of pneumococcal serotype 1 (Table 2). In this region, most reported STs belonged to clonal complex 306. In Australia, ST304 and ST227 were widely reported in serotype 1 IPD cases and carriage in the north, according to Smith-Vaughan et al. [47]. An outbreak of serotype 1 IPD in Australia between 2008 and 2012 was predominantly caused by ST306 clones [51,53]. The proportion of ST304 isolates reported in Australia significantly decreased during this period, attributed to the clonal expansion of CC306, characterized by an increase in ST306 isolates (Figure 8). In New Caledonia (NC), ST306 emerged as the most predominant serotype 1 clone responsible for outbreaks reported [38]. Genomic characterization of serotype 1 isolates from outbreaks in NC discovered ST306 strains (62/67, 92.53%) and their single locus variants, ST3717 (5/67, 7.46%) (Figure 8) [38]. Additionally, ST306 serotype 1 isolates were reported in cases of IPD in New Zealand. Figure 4 highlights the dominant clones found in this region.

## 3. Discussion

*Streptococcus pneumonaie* serotype 1 has been a leading cause of severe pneumococcal disease worldwide, affecting both children and adults. Notably, this serotype is seldom found in healthy individuals, suggesting that while it may not be easily transmitted, it has a high tendency to progress to invasive disease once carried. This implies that serotype 1 is remarkably hypervirulent, corroborating its frequent association with outbreaks of severe pneumococcal infection. Furthermore, several clones, including ST217 and ST306, have been linked to outbreaks of IPD globally. In light of these findings, we conducted a systematic review to elucidate the molecular epidemiology of *S. pneumoniae* serotype 1 clones circulating worldwide. This comprehensive analysis seeks to identify high-risk clones with the potential to trigger outbreaks in various countries or continents, ultimately informing targeted evidence-based strategies for disease prevention and control.

The multilocus sequence typing (MLST) technique is a widely accepted method for bacterial genotyping, leveraging seven housekeeping genes (*aroE*, *gdh*, *gki*, *recP*, *spi*, *xpt*, and *ddl*) to characterize *S. pneumoniae* isolates. As the golden standard for *S. pneumoniae* genotyping, MLST was mainly used in most studies to elucidate the genetic diversity of this pathogen. We identified one hundred and three (103) different MLST sequence types associated with serotype 1. Most of these STs shared genetic relationships with ST217, followed by ST306.

The *S. pneumoniae* ST217 clonal complex is a hypervirulent lineage of significant public health concern. In our study, this lineage was mostly reported in studies from Africa and Asia. The ancestral clone, ST217, was predominantly associated with pneumococcal meningitis across several countries, including Nigeria, Niger, Israel, India, Thailand, Singapore, and the Philippines. ST217 was also linked to numerous IPD cases in Kenya, South Africa, Ethiopia, Mozambique, and Malawi. Although not the most common clone, ST217 was associated with a serotype 1 pneumococcal meningitis outbreak in northern Ghana [50]. This finding is not surprising since the predominance of ST217 in sub-Saharan African countries has been previously reported in several studies [23]. This widespread presence of ST217 may be attributed to intra and intercontinental movements of people between countries, leading to the transfer of clones. Other clones within CC217, such as ST618, were prevalent among serotype 1 isolates from both invasive and carriage isolates in Gambia. Also, ST618 was responsible for pneumococcal meningitis in patients from Burkina Faso [64] and Egypt [74]. Additionally, studies from neighboring countries like Ghana and Togo, as well as Nepal and Bangladesh, mostly described ST303 as associated with bacterial meningitis. Several other STs within CC217 were also identified in Africa and Asia indicating an extensive clonal expansion and diversity of the ST217 clonal complex, particularly in Africa. The propensity of ST217 and its clonal complex 217 to cause pneumococcal meningitis poses a significant threat to these regions, emphasizing the importance of implementing mitigation strategies to curb their spread. Fortunately, many isolates of CC217 are sensitive to antibiotics, making their treatment easy and effective [50]. While the ST217 clonal complex is predominantly reported in Africa and Asia, its presence has also been noted in other parts of the world. For example, studies have reported STs from the clonal complex in regions like Australia, the US, and specific countries in Europe.

ST306 clonal complex is another lineage of serotype 1 of public health importance. This group emerged as the prominent group in Europe, Oceania, North America, and some countries in South America characterized by its division into three main subgroups: ST306, ST304, and ST227. ST306 (ancestral clone) was the most predominant ST among this group widely dispersed in most European countries. ST304 was common in Mexico, Brazil, Uruguay, and Slovenia. STs of CC306 were more associated with bacteremia than other pneumococcal diseases. Our analysis revealed that ST227 was the predominant clone in Canada and the US, supporting the findings of Brueggemann & Spratt [44]. However, our results diverged from theirs regarding the UK, where they reported ST227 as the dominant clone. This discrepancy suggests that a clonal shift may have occurred in the UK over time, potentially replacing ST227 with ST306, as discovered in our study. This aligns with findings reported by Pinchon et al. [75] in 2013, revealing a reduced proportion of ST227 and an increased prevalence of ST306, representing 83% of serotype 1 meningitis cases in the UK. Although all serotype 1 clones are hypervirulent, a modification in one’s genetic material may enhance its invasiveness, possibly explaining the observed clonal shift [75]. Like CC217, the genetic variation of ST306 clonal complex is also illustrated by the identification of several other related STs.

One key finding of our study is the geographic structure among major serotype 1 lineages, revealing distinctive regional differences in circulating clones. This observation may be attributed to the rarity of serotype 1 carriage. Unlike other serotypes that can easily be carried and transferred between populations via human movement, the rarity of serotype 1 carriage restricts its geographic spread and may contribute to unique clonal distributions. Also, this unique clonal distribution may be attributed to geographic weather conditions in the various regions. For instance, it might be that the ST217 clonal complex thrives in humid and tropical areas, which may explain why they are widely found in Africa and Asia. This possibly applies to ST306 and ST615 clonal complexes that are common in temperate climate areas.

While our study provides valuable insight into the global circulation of serotype 1 *S. pneumoniae*, it is not without limitations. One notable constraint is the geographical representation of the included studies, predominantly clustered in forty-eight (48) countries. The uneven distribution leaves a significant knowledge gap in many countries, highlighting the need for further research to understand better the epidemiological landscape of pneumococcal serotype 1 clones in these underrepresented regions.

## 4. Materials and Methods

This systematic review adhered to the Preferred Reporting Items for Systematic Reviews and Meta-Analyses (PRISMA) guidelines [76].

### 4.1. Literature Search Strategy and Selection Process

An extensive literature search was conducted in Google Scholar, PubMed, Scopus, ScienceDirect, and Web of Science using keywords related to *Streptococcus pneumoniae* serotype 1. The search was conducted until October 2024 and restricted to free full-text or open-access research articles published in English (Table 3). Retrieved records were exported in RIS and CSV formats.

Citations of the retrieved records were imported to the Rayyan application [77] (a web-based tool for systematic reviews) for screening. Duplicate records were first detected and removed, followed by title and abstract screening of the remaining unique records, where all unrelated records were excluded. After title and abstract screening, the remaining articles were assessed for full-text eligibility based on the abovementioned inclusion criteria. Included studies were imported to Zotero 7.0.8 for data extraction. O.K.N. and E.S.D. assessed the eligibility of the retrieved records.

### 4.2. Eligibility Criteria

This study included English-published peer-reviewed reports that utilized at least one molecular genotyping tool to describe *S. pneumoniae* serotype 1 clones based on their sequence types (STs). Multicenter studies describing serotype 1 clones in specific geographic regions (e.g., West Africa, sub-Saharan Africa) or those reporting clone data for individual countries were eligible for inclusion. Exclusions included non-peer-reviewed sources (Theses, dissertations, editorials, comments, preprints, etc.), studies not reporting serotype 1 clones, studies that selected specific serotype 1 clones for genetic analysis, and studies that focused or reported on other serotypes or their clones.

### 4.3. Data Extraction and Analysis

The relevant data were extracted from each included study as described in Table 2 by O.K.N. and E.S.D. The sequence types (STs) of *S. pneumoniae* serotype 1 clones were extracted and stratified by country/region. Microsoft Excel 365 (Version 2108) was used to extract and manage the extracted data.

### 4.4. BURST Cluster Analysis

The allelic profiles of identified STs were downloaded from the *Streptococcus pneumoniae* database on the MLST website https://pubmlst.org/organisms/streptococcus-pneumoniae (accessed on 15 November 2024). STs were grouped into clonal complexes according to their allelic profiles using the BURST algorithm [78]. Furthermore, the geoBURST algorithm in the Phyloviz online application (https://online.phyloviz.net/index, accessed on 15 November 2024) was used to construct the minimum spanning tree, providing a visual representation of the genetic relationship among the STs.

### 4.5. Risk of Bias Assessment

Two investigators (O.K.N. and E.S.D.) assessed the risk of bias of the included studies using the STROBE checklist for reporting observational studies [79], as adapted according to Sanderson et al. [80]. The assessment tool comprised five key domains: method of selecting study participants, method of measuring exposure and outcome variables, method of control confounding, design-specific source of bias, and statistical methods (Supplementary Table S2). Studies were graded into three categories: High risk of bias (≥1 of any criteria classified as H, or ≥2 major criteria as M or U); Moderate risk of bias (≥2 of any criteria classified as M or U [<2 major criteria]); Low risk of bias (all major criteria classified as L and <2 minor criteria as M or U).

## 5. Conclusions

This systematic review highlights the complex genetic diversity of *S. pneumoniae* serotype 1, characterized by distinct geographic structures among major lineages. A striking continental distribution emerged, with the ST217 clonal complex predominantly clustered in Africa and Asia, whereas the ST306 clonal complex was widespread across Europe, the Americas, and Oceania. Notably, IPD was primarily linked to clones from these clonal complexes, with few instances of carriage. The findings of this study emphasize the need for enhanced surveillance and targeted interventions to mitigate the spread of hypervirulent clones, ultimately informing evidence-based strategies for disease prevention and control. To address existing knowledge gaps, further research is warranted, particularly in countries with limited or no data on serotype 1 *S. pneumoniae* clones.

## Figures and Tables

**Table 1:** Characteristics of forty-three included studies.

Author, Year [Ref]	Country	Year of Study	Study Population	Study Group	Isolate Type	No. of *S. Pneumoniae* Serotype 1	Molecular Identification Method	Genotyping Tools	
								MLST	WGS
Byington et al., 2010 [27]	United States	2001–2007	Children with Pediatric pneumococcal empyema	Children	IPD	17	Unspecified	√	-
Chaguza et al., 2020 [37]	sub-Saharan Africa	1996–2016	Invasive pneumococcal serotype 1 isolates from sub-Saharan Africa	All ages	IPD	909	Whole genome sequencing	√	√
Hanachi et al., 2020 [38]	New Caledonia	2004–2009	Pneumococcal serotype 1 samples	Unspecified	IPD & NIPD	67	Whole genome sequencing	√	√
Almeida et al., 2013 [39]	Portugal	1996–2010	Healthy children attending daycare	Children	Carriage	21	PCR	√	-
Esteva et al., 2011 [40]	Spain	1989–2008	Children with IPD	Children	IPD	56	Unspecified	√	-
Marimon et al., 2009 [41]	Spain	1987–2007	Pneumococcal serotype 1 samples	All ages	IPD & NIPD	135	Unspecified	√	-
Antonio et al., 2008 [42]	Gambia	1996–2006	Healthy Gambians and IPD cases	All ages	IPD & Carriage	163	PCR	√	-
Gonzalez et al., 2004 [43]	United States	1993–2002	Pediatric patients	Children	IPD & NIPD	55	Rep-PCR	√	-
Brueggemann & Spratt, 2003 [44]	Multicenter	1990–2001	Serotype 1 IPD isolates	All ages	IPD	166	PCR	√	-
Jourdian et al., 2013 [45]	Belgium	2006–2008	Nursery school children	Children, 3–6 years	Carriage	11	PCR	√	-
Horacia et al., 2016 [46]	Portugal	2008–2011	Adult patients with IPD	Adults, 18 years and above	IPD	66	Unspecified	√	-
Smith-Vaughan et al., 2009 [47]	Australia	1992–2007	Individuals in remote Indigenous communities in Australia	All ages	IPD & Carriage	26	PCR	√	-
Jauneikaite et al., 2014 [48]	Singapore	2009–2010	IPD cases	Unspecified	IPD	4	PCR	√	-
Kwambana-Adams et al., 2016 [49]	Ghana		Cases of suspected meningitis	All ages	IPD	38	Triplex qPCR	√	-
Leimkugel et al., 2005 [50]	Ghana	1998–2003	Patients with suspected meningitis	All ages	IPD	58	PCR	√	-
Lai et al., 2013 [51]	Australia	2010–2012	participants presenting to or visiting the Alice Springs Hospital Emergency Department	All ages, 1–77 years	Carriage	4	multiplex PCR	√	-
Ebruke et al., 2015 [52]	Gambia	2003–2004	Healthy Gambians	All ages	Carriage	81	multiplex PCR	√	-
Kirkham et al., 2006 [22]	United Kingdom	2000–2004	Serotype 1 IPD isolates	All ages	IPD	34	PCR	√	-
Staples et al., 2014 [53]	Australia	2008–2012	Serotype 1 IPD isolates	Unspecified	IPD	253	PCR	√	-
Zhou et al., 2017 [36]	China	2006–2012	Patients in a hospital in Shanghai, China	Children, <10 years	IPD & NIPD	15	PCR	√	-
du Plessis et al., 2016 [54]	South Africa	1989–2013	IPD cases	All ages	IPD	534	Whole genome sequencing	√	√
Brueggemann et al., 2013 [55]	Kenya	1994–2008	Healthy persons and ill children	Children, <15 years	IPD & Carriage	161	PCR	√	-
Donkor et al., 2013 [56]	West Africa	1996–2007	Pneumococcal carriage and IPD isolates	Children, <15 years	IPD & Carriage	7	PCR	√	-
Zemlicková et al., 2005 [57]	Multicenter	1990–2002	Children with IPD	Children, <5 years	IPD	26	PCR	√	-
Muñoz-Almagro et al., 2011 [58]	Spain	2009	Patients with IPD	All ages	IPD	137	multiplex PCR	√	-
Cooke et al., 2010 [59]	United Kingdom	1999–2007	IPD cases	Unspecified	IPD	225	Unspecified	√	-
Clarke et al., 2006 [60]	United Kingdom	2000–2004	Children with IPD	Children, <5 years	IPD	7	Unspecified	√	-
Muñoz-Almagro et al., 2008 [61]	Spain	1997–2006	Patients with IPD	All ages	IPD	34	Unspecified	√	-
Beall et al., 2006 [62]	United States	1999–2002	IPD cases	Unspecified	IPD	63	Unspecified	√	-
Antonio, Dada-Adegbola, et al., 2008 [63]	Gambia	2000–2004	Children investigated for possible IPD	Children, 2–29 months	IPD	8	Box PCR	√	-
Yaro et al., 2006 [64]	Burkina Faso	2002–2005	Persons with suspected acute bacterial meningitis	All ages, 2–29 years	IPD	21	PCR	√	-
Zahner et al., 2010 [65]	United States	1994–2006	IPD cases	Unspecified	IPD	5	Unspecified	√	-
Grau et al., 2012 [28]	Spain	1996–2010	Patients with IPD	Adults, 18–64 years	IPD	76	PCR	√	-
Kourna Hama et al., 2019 [66]	Niger	2010–2016	Children with suspected meningitis	Children, <5 years	IPD	10	qPCR, Whole genome sequencing	-	√
Jefferies et al., 2010 [67]	United Kingdom	2001–2006	IPD cases	All ages	IPD	261	PCR	√	-
Porat et al., 2012 [68]	Israel	1999–2008	Cases of AOM & IPD	Children	IPD & NIPD	92	Unspecified	√	-
Foster et al., 2008 [69]	United Kingdom	1996–2005	IPD cases	All ages	IPD	203	Unspecified	√	-
Chaguza et al., 2017 [70]	Malawi	2004–2010	IPD cases	All ages	IPD	113	PCR, whole genome sequencing	√	√
Sanneh et al., 2019 [71]	Gambia	2010–2016	Suspected cases of meningitis	Children, <5 years	IPD	17	qPCR, multiplex PCR, Whole genome sequencing	-	√
Tsolenyanu et al., 2019 [72]	Togo	2010–2016	Suspected cases of meningitis	Children, <5 years	IPD	7	RT-PCR, whole genome sequencing	-	√
Sharew et al., 2024 [35]	Ethiopia	2018–2019	Patients with IPD and NIPD	All ages	IPD & NIPD	1	Whole genome sequencing	√	√
Serrano et al., 2005 [73]	Portugal	1999–2002	IPD cases	Children	IPD	470	Unspecified	√	-
Cornick et al., 2015 [74]	Multicenter	2003–2008	serotype 1 pneumococci recovered from hospital, surveillance, and carriage studies	All ages	IPD, NIPD & Carriage	448	Whole genome sequencing	√	√

IPD: invasive pneumococcal disease; NIPD: non-invasive pneumococcal disease; PCR: polymerase chain reaction; qPCR: real-time polymerase chain reaction; AOM: acute otitis media.

**Table 2:** Number of studies and sequence types reported in the various continents.

Continent	Countries	No. Studies	No. STs				
			CC217	CC306	CC615	Singletons	Others
Africa	Gambia, Ghana, Burkina Faso, Egypt, Ethiopia, Kenya, Malawi, Mozambique, Niger, Nigeria, South Africa, Senegal, Togo	17	48	2	1	13	1
Europe	Belgium, Croatia, Czech Republic, Denmark, France, Germany, Netherlands, Norway, Slovenia, Sweden, United Kingdom, Poland, Portugal, Spain	16	3	12	3	1	-
Asia	Bangladesh, China, India, Israel, Nepal, Philippines, Qatar, Singapore, Thailand	5	9	2	3	7	1
North America	Canada, Mexico, United State	6	2	8	1	1	-
South America	Argentina, Brazil, Chile, Colombia, Peru, Uruguay	3	-	2	1	-	-
Oceania	Australia, New Caledonia, New Zealand	6	3	4	1	1	-

CC: clonal complex; ST: sequence type.

**Table 3:** Detailed search keywords and filters used in each database.

Database	Keywords	Filters		
		Language	Document Type	Open Access
PubMed	“*Streptococcus pneumoniae* serotype 1” OR “serotype 1 *Streptococcus pneumoniae*” OR “pneumococcal serotype 1”	English	-	Free full-text
Scopus		English	Article	All Open Access
Web of Science		English	Article	Open Access
ScienceDirect		English	Research article	Open Access & Open archives
Google Scholar		English	-	-

## Data Availability

Data and materials used for the analysis are available upon request. Tables S1 and S2 are available as Supplementary Materials.

## Supplementary Materials

The following supporting information can be downloaded at: https://www.mdpi.com/article/10.3390/ijms26052266/s1. References [81,82] are cited in the supplementary materials.
