# Supplementary material for: Molecular Epidemiology of Streptococcus pneumoniae Serotype 1: A Systematic Review of Circulating Clones and Clonal Clusters
Source: Int J Mol Sci. 2025 Mar 4;26(5):2266. doi: 10.3390/ijms26052266 (PMC11900055; doi:10.3390/ijms26052266)
Supplement: Supplementary file 1 [file ijms-26-02266-s001.zip › Supplementary_tables.pdf]

Table S1. Molecular characteristics of 103 sequences types found in this study.

Clonal Complex	Sub-CC	ST	Allelic profile							Location(s) where ST was reported (No. of isolates) (ref)
			aroE	gdh	gki	recP	spi	xpt	ddl	
217	217	217*	10	18	4	1	7	19	9	Ethiopia (3) [1,2], Gambia (17) [1,3–5], Ghana (18) [1,6], Malawi (60) [1,7], Mozambique (41) [1], Niger (31) [1,8], Nigeria (2) [1], South Africa (436) [1,9,10], , Togo (1) [11], US (2) [12], Kenya (114) [9,13], Belgium (1) [14], Denmark (1) [9], France (2) [9], Spain (2) [15,16] Germany (2) [9], UK (1) [17], Thailand (13) [1], Israel (11) [9,18], India (4) [1], Philippines (1) [1], Qatar (1) [1], Singapore (1) [19]
		613	10	18	4	1	7	19	1	Kenya (28) Brueggemann et al., 2013; Brueggemann & Spratt, 2003
		614	10	18	4	1	3	19	9	Kenya (29) [9,13], Spain (2) [20]
		1316	2	18	4	1	7	19	9	Ghana (1) [6]
		1325	10	8	4	1	7	19	9	Ghana (2) [6]
		2034	10	18	4	1	7	19	17	Egypt (1) [1]
		2206	10	133	4	1	7	19	9	Niger (2) [1]
		2565	7	18	4	1	7	19	9	Australia (1) [21]
		2839	13	18	4	1	7	19	9	India (1) [1], Niger (1) [1]
		3079	10	18	4	1	7	19	276	Australia (2) [22]
		3575	10	191	4	1	7	19	9	Gambia (12) [3]
		5012	10	18	4	1	6	19	9	Sub-Saharan Africa [23]
		5254	10	18	4	1	7	393	9	Singapore (1) [19]
		5632	10	18	4	1	7	351	9	Sub-Saharan Africa [23]
		8158	10	10	4	1	7	19	9	Malawi (1) [1]
		8314	10	18	36	1	7	19	9	South Africa (8) [24]
		9067	10	18	4	1	7	525	9	South Africa (16) [24]
		9529	10	18	4	1	7	83	9	Sub-Saharan Africa [23]
		10649	10	338	4	1	7	19	9	Sub-Saharan Africa [23]
		11743	10	453	4	1	7	19	9	Sub-Saharan Africa [23]
		11745	10	464	4	1	7	19	9	Sub-Saharan Africa [23]
		12150	379	18	4	1	7	83	9	Sub-Saharan Africa [23]
		12165	381	18	4	1	7	19	9	Sub-Saharan Africa [23]
		12333	10	18	4	8	7	19	9	Sub-Saharan Africa [23]
		12546	10	18	4	1	7	19	791	Sub-Saharan Africa [23]
		12556	10	18	4	1	7	19	782	Sub-Saharan Africa [23]
		12739	10	18	553	1	541	19	9	Sub-Saharan Africa [23]
	618	618	13	8	4	1	7	19	14	Burkina Faso [25], Egypt (3) [1], Gambia (158) [1,3–5,26], South Africa (17) [10], Togo (3) [11], Spain (2) [20], Netherland (1) [9]
		1331	13	8	4	1	7	19	9	Gambia (2) [3], Ghana (2) [6]

		2084	13	8	4	2	7	19	14	Gambia (5) [3], Ghana (2) [6]
		3336	13	8	4	1	7	19	161	Gambia (1) [26]
		3573	13	8	4	15	7	19	14	Gambia (1) [3]
		3574	13	8	4	1	7	19	19	Gambia (3) [3]
		3577	10	8	4	1	7	19	14	Gambia (1) [3]
		3579	13	8	4	5	7	250	14	Gambia (1) [3]
		3581	13	8	4	115	7	19	14	Gambia (2) [3]
		5044	13	16	4	1	7	19	17	India (3) [1], Singapore (1) [19]
	303	303	10	5	4	1	7	19	9	Gambia (7) [1,4,5], Ghana (70) [1,6,27], Malawi (2) [1], Niger (15) [1], Togo (9) [1,11], Bangladesh (7) [1], Qatar (4) [1], Singapore (1) [19], Thailand (4) [1], Nepal (4) [1], US (1) [9]
		1322	10	5	4	1	7	19	31	Ghana (1) [6]
		1323	10	5	4	1	7	21	9	Ghana (1) [6]
		4755	10	5	4	1	7	323	9	Ghana (2) [1]
		5002	10	5	4	1	17	19	9	India (1) [1]
		5672	179	5	4	1	17	19	9	India (3) [1]
		11104	10	5	226	1	7	19	9	Sub-Saharan Africa [23]
		11736	10	5	4	1	7	19	683	Sub-Saharan Africa [23]
		12305	10	5	4	1	7	483	9	Sub-Saharan Africa [23]
		12803	10	5	4	1	7	734	9	Sub-Saharan Africa [23]
	612	612	10	18	4	1	7	19	31	Gambia (4) [3], Ghana (8) [6], South Africa (113) [1,9,10], Israel (1) [9], Australia (1) [21]
		1327	10	18	4	1	13	19	31	Ghana (1) [6]
		1328	10	18	4	1	7	21	31	Ghana (1) [6]
		3570	7	18	4	1	7	19	31	Gambia (1) [3]
	3081	3081	10	18	4	1	7	232	9	Gambia (93) [1,4,5], Philippines (1) [1], Senegal (4) [1]
		11779	338	18	4	1	7	232	9	Sub-Saharan Africa [23]
		12197	388	18	4	1	7	232	9	Sub-Saharan Africa [23]
306	306	306*	12	8	13	5	16	4	20	Belgium (7) [14], Croatia (1) [1], Czech Republic (2) [28], Denmark (6) [9], France (3) [9], Germany (8) [9], Netherland (3) [9], Norway (8) [9], Poland (5) [9], Portugal (77) [29–31], Sweden (14) [1], UK (246) [17,32–34], Spain (315) [1,9,15,16,20,35,36], South Africa (4) [10], Qatar (1) [1], US (6) [9,37], Canada (1) [9], Colombia (5) [28], Australia (246) [21,38], New Caledonia (62) [39]
		228	12	8	1	5	17	4	20	Croatia (1) [1], France (2) [9], Netherland (3) [9], Portugal (3) [29,31], Spain (34) [1,15,16,20,36], Canada (3) [9]
		617	12	64	13	5	16	4	20	Norway (1) [9]
		1247	12	8	13	5	6	111	20	United Kingdom (1) [17]
		1310	12	8	13	5	16	4	14	United Kingdom (1) [17]
		2376	12	8	7	5	16	4	20	Spain (2) [15,36]
		3717	124	8	13	5	16	4	20	New Caledonia (5) [39]
		4290	12	8	13	5	16	4	8	United States (2) [37]

304	304	13	8	13	5	17	4	8	South Africa (1) [10], Belgium (1) [14], France (3) [9], Netherland (3) [9], Norway (1) [9], Portugal (2) [29,31], Slovenia (1) [1], Spain (55) [1,9,15,20,35,36], UK (152) [32,40], Israel [18], Argentina (2) [28], Brazil (13) [1,28], Canada (1) [9], US (5) [9,37,41], Uruguay (4) [28], Mexico (4) [28], Australia (10) [21,22], New Zealand (3) [1]
	305	13	8	13	5	17	4	28	Denmark (1) [9], Netherland (2) [9], Spain (2) [16]
	350	13	8	13	5	13	4	28	Belgium (2) [14], Portugal (5)[31,42]
	1174	13	8	13	10	17	4	8	United States (2) [12]
	3861	13	10	13	5	17	4	8	Spain (1) [16]
	4288	13	8	13	5	209	7	8	United States (4) [37]
227	227	12	5	13	5	17	4	20	UK (115) [9,17,32,34,40], Spain (15) [9,16], Netherland (1) [9], Denmark (1) [9], US (124) [9,12,37,41,43], Canada (15) [9], Australia (6) [22]
	2126	12	5	13	5	16	4	20	United States (1) [37]
	1239	12	5	13	5	16	1	20	United Kingdom (1) [17]
615	300	10	31	4	34	6	4	5	United Kingdom [9,34]
	611	10	9	4	1	6	4	94	South Africa (7) [9,24]
	615*	10	31	4	1	6	4	94	Thailand (8) [1], Argentina (8) [1,28], Chile (11) [9], Peru (1) [1], US (1) [9], Uruguay (1) [28]
	616	10	31	4	1	62	4	94	Poland (2) [9]
	3018	10	31	4	16	6	4	94	Australia (1) [21], Qatar (2) [1]
	2296	10	31	4	34	6	4	94	China (7) [1]
	3860	10	5	4	1	6	4	8	Spain (1) [16]
Others	802	10	13	53	1	72	38	31	China (1) [44]
	3580	10	13	53	1	72	19	31	Gambia (1) [3]
Singletons	81	4	4	2	4	4	1	1	China (5) [44]
	162	7	11	10	1	6	8	14	United Kingdom (1) [32]
	242	15	29	4	21	30	1	14	China (3) [44]
	342	7	32	8	12	6	6	63	China (3) [44]
	632	2	8	2	4	6	75	1	Mozambique (1) [1]
	1263	15	13	4	16	6	1	17	China (1) [44]
	1336	1	87	9	2	6	48	6	Gambia (1) [3]
	3044	5	109	1	2	6	1	14	India (3) [1]
	3329	7	13	4	8	6	20	18	Gambia (1) [3]
	3397	7	187	122	87	17	1	290	China (1) [44]
	3407	23	189	4	12	43	4	74	Gambia (1) [3]
	3565	2	19	2	17	6	22	15	West Africa (1) [45]
	3571	7	13	101	4	36	1	74	Gambia (1) [3]
	3572	7	2	4	4	7	17	19	Gambia (1) [3]
	3576	5	17	4	4	6	1	17	Gambia (1) [3]

3578	1	8	193	5	6	58	8	Gambia (1) [3]
3582	12	43	194	1	181	49	31	Gambia (1) [3]
3583	2	20	4	38	27	88	6	Gambia (1) [3]
3960	13	1	8	1	7	38	14	West Africa (1) [45]
4289	13	14	14	5	17	4	20	United States (1) [37]
7712	234	9	111	16	327	329	8	Mozambique (1) [1]
9072	8	9	2	1	6	28	17	Australia (1) [21]
13362	4	4	2	4	6	20	26	China (1) [44]

Table S2. Risk of bias assessment of included studies

Study	Methods for selecting study participants	Methods for measuring exposure and outcome variables	Methods to control confounding	Design-specific sources of bias	Statistical methods	Risk of bias
Byington et al., 2009	L	M	M	H	L	High
Chaguza et al., 2020	U	L	L	L	M	Low
Hanachi et al., 2020	L	L	L	M	L	Low
Almeida et al., 2013	U	L	M	M	L	High
Esteva et al., 2011	L	U	M	M	L	Moderate
Marimon et al., 2009	M	M	M	M	M	Moderate
Antonio et al., 2008	L	U	M	M	L	Moderate
Gonzalez et al., 2004	L	M	M	M	L	High
Brueggemann et al., 2003	L	M	L	M	L	Moderate
Jourdian et al., 2013	M	L	M	L	L	Moderate
Horacia et al., 2016	L	M	M	M	L	High
Smith-Vaughan et al., 2008	L	M	M	M	M	High
Jauneikaite et al., 2014	L	M	M	M	L	Moderate
Kwambana-Adams et al., 2016	L	L	L	M	L	Low
Leimkugel et al., 2005	L	H	L	M	L	High
Lai et al., 2013	M	L	M	M	L	Moderate
Ebruke et al., 2015	L	L	M	M	L	Moderate
Kirkham et al., 2006	L	M	L	H	L	High

Staples te al., 2014	L	L	L	M	L	Low
Zhou et al., 2017	L	L	L	U	M	Low
Du Plessis et al., 2016	L	M	M	M	L	High
Brueggemann et al., 2013	M	L	M	L	L	High
Donkor et al., 2013	L	M	M	M	L	Moderate
Zemlickova et al, 2005	L	L	L	M	L	Low
Munoz-almgro et al., 2011	L	L	L	U	M	Low
Cooke et al., 2010	L	M	M	M	L	Moderate
Clarke et al., 2006	L	M	M	L	L	High
Munoz-Almagro et al., 2008	M	M	M	M	L	Moderate
Beall et al., 2006	L	M	L	M	L	High
Antonio et al., 2008	L	L	L	U	L	Low
Yaro et al., 2006	L	M	M	M	L	High
Zahner et al., 2010	L	U	M	M	M	Moderate
Grau et al., 2012	L	H	L	M	L	High
Kourna Hama et al., 2019	L	L	L	M	L	Low
Jefferies et al., 2009	L	L	L	M	L	Low
Porat et al., 2012	L	M	L	M	L	Moderate
Foster et al., 2008	L	H	M	M	L	Moderate
Chaguza et al., 2017	L	M	M	M	L	Moderate
Sanneh et al., 2019	L	H	L	M	L	High
Tsolenyanu et al., 2019	M	L	M	M	L	Moderate
Sharew et al., 2024	L	L	M	M	L	High
Serrano et al., 2005	L	M	L	M	L	Moderate
Cornick et al., 2015	L	M	M	M	M	Moderate

References

1. Cornick JE, Chaguza C, Harris SR, Yalcin F, Senghore M, Kiran AM, et al. Region-specific diversification of the highly virulent serotype 1 Streptococcus pneumoniae. Microb Genomics. 2015 Aug;1(2):e000027.

2. Sharew B, Moges F, Yismaw G, Mihret A, Lobie TA, Abebe W, et al. Molecular epidemiology of Streptococcus pneumoniae isolates causing invasive and noninvasive infection in Ethiopia. Sci Rep. 2024 Sep 13;14(1):21409.

3. Antonio M, Hakeem I, Awine T, Secka O, Sankareh K, Nsekpong D, et al. Seasonality and outbreak of a predominant Streptococcus pneumoniae serotype 1 clone from The Gambia: expansion of ST217 hypervirulent clonal complex in West Africa. BMC Microbiol. 2008 Nov 17;8:198.

4. Ebruke C, Roca A, Egere U, Darboe O, Hill PC, Greenwood B, et al. Temporal changes in nasopharyngeal carriage of Streptococcus pneumoniae serotype 1 genotypes in healthy Gambians before and after the 7-valent pneumococcal conjugate vaccine. PeerJ. 2015;3:e903.

5. Sanneh B, Okoi C, Grey-Johnson M, Bah-Camara H, Kunta Fofana B, Baldeh I, et al. Declining Trends of Pneumococcal Meningitis in Gambian Children After the Introduction of Pneumococcal Conjugate Vaccines. Clin Infect Dis Off Publ Infect Dis Soc Am. 2019 Sep 5;69(Suppl 2):S126–32.

6. Leimkugel J, Adams Forgor A, Gagneux S, Pflüger V, Flierl C, Awine E, et al. An outbreak of serotype 1 Streptococcus pneumoniae meningitis in northern Ghana with features that are characteristic of Neisseria meningitidis meningitis epidemics. J Infect Dis. 2005 Jul 15;192(2):192–9.

7. Chaguza C, Cornick JE, Andam CP, Gladstone RA, Alaerts M, Musicha P, et al. Population genetic structure, antibiotic resistance, capsule switching and evolution of invasive pneumococci before conjugate vaccination in Malawi. Vaccine. 2017 Aug 16;35(35 Pt B):4594–602.

8. Kourna Hama M, Khan D, Laouali B, Okoi C, Yam A, Haladou M, et al. Pediatric Bacterial Meningitis Surveillance in Niger: Increased Importance of Neisseria meningitidis Serogroup C, and a Decrease in Streptococcus pneumoniae Following 13-Valent Pneumococcal Conjugate Vaccine Introduction. Clin Infect Dis Off Publ Infect Dis Soc Am. 2019 Sep 5;69(Suppl 2):S133–9.

9. Brueggemann AB, Spratt BG. Geographic distribution and clonal diversity of Streptococcus pneumoniae serotype 1 isolates. J Clin Microbiol. 2003 Nov;41(11):4966–70.

10. du Plessis M, Allam M, Tempia S, Wolter N, de Gouveia L, von Mollendorf C, et al. Phylogenetic Analysis of Invasive Serotype 1 Pneumococcus in South Africa, 1989 to 2013. J Clin Microbiol. 2016 May;54(5):1326–34.

11. Tsolenyanu E, Bancroft RE, Sesay AK, Senghore M, Fiawoo M, Akolly D, et al. Etiology of Pediatric Bacterial Meningitis Pre- and Post-PCV13 Introduction Among Children Under 5 Years Old in Lomé, Togo. Clin Infect Dis Off Publ Infect Dis Soc Am. 2019 Sep 5;69(Suppl 2):S97–104.

12. Beall B, McEllistrem MC, Gertz RE, Wedel S, Boxrud DJ, Gonzalez AL, et al. Pre- and postvaccination clonal compositions of invasive pneumococcal serotypes for isolates collected in the United States in 1999, 2001, and 2002. J Clin Microbiol. 2006 Mar;44(3):999–1017.

13. Brueggemann AB, Muroki BM, Kulohoma BW, Karani A, Wanjiru E, Morpeth S, et al. Population genetic structure of Streptococcus pneumoniae in Kilifi, Kenya, prior to the introduction of pneumococcal conjugate vaccine. PloS One. 2013;8(11):e81539.

14. Jourdain S, Drèze PA, Verhaegen J, Van Melderen L, Smeesters PR. Carriage-associated Streptocccus pneumoniae serotype 1 in Brussels, Belgium. Pediatr Infect Dis J. 2013 Jan;32(1):86–7.

15. Esteva C, Selva L, de Sevilla MF, Garcia-Garcia JJ, Pallares R, Muñoz-Almagro C. Streptococcus pneumoniae serotype 1 causing invasive disease among children in Barcelona over a 20-year period (1989-2008). Clin Microbiol Infect Off Publ Eur Soc Clin Microbiol Infect Dis [Internet]. 2011 Sep [cited 2024 Nov 4];17(9). Available from: https://pubmed.ncbi.nlm.nih.gov/21729192/

16. Marimon JM, Ercibengoa M, Alonso M, Zubizarreta M, Pérez-Trallero E. Clonal structure and 21-year evolution of Streptococcus pneumoniae serotype 1 isolates in northern Spain. Clin Microbiol Infect Off Publ Eur Soc Clin Microbiol Infect Dis [Internet]. 2009 Sep [cited 2024 Nov 4];15(9). Available from: https://pubmed.ncbi.nlm.nih.gov/19702591/

17. Kirkham LAS, Jefferies JMC, Kerr AR, Jing Y, Clarke SC, Smith A, et al. Identification of Invasive Serotype 1 Pneumococcal Isolates That Express Nonhemolytic Pneumolysin. J Clin Microbiol. 2006 Jan;44(1):151.

18. Porat N, Benisty R, Trefler R, Givon-Lavi N, Dagan R. Clonal distribution of common pneumococcal serotypes not included in the 7-valent conjugate vaccine (PCV7): marked differences between two ethnic populations in southern Israel. J Clin Microbiol. 2012 Nov;50(11):3472–7.

19. Jauneikaite E, Jefferies JM, Churton WV, Lin RPT, Hibberd ML, Clarke CS. Genetic diversity of Streptococcus pneumoniae causing meningitis and sepsis in Singapore during the first year of PCV7 implementation. Emerg Microbes Infect [Internet]. 2014 Jun [cited 2024 Nov 4];3(6). Available from: https://pubmed.ncbi.nlm.nih.gov/26038742/

20. Muñoz-Almagro C, Ciruela P, Esteva C, Marco F, Navarro M, Bartolome R, et al. Serotypes and clones causing invasive pneumococcal disease before the use of new conjugate vaccines in Catalonia, Spain. J Infect. 2011 Aug;63(2):151–62.

21. Staples M, Graham RMA, Jennison AV, Ariotti L, Hicks V, Cook H, et al. Molecular characterization of an Australian serotype 1 Streptococcus pneumoniae outbreak. Epidemiol Infect. 2015 Jan;143(2):325–33.

22. Smith-Vaughan H, Marsh R, Mackenzie G, Fisher J, Morris PS, K H, et al. Age-specific cluster of cases of serotype 1 Streptococcus pneumoniae carriage in remote indigenous communities in Australia. Clin Vaccine Immunol CVI [Internet]. 2009 Feb [cited 2024 Nov 4];16(2). Available from: https://pubmed.ncbi.nlm.nih.gov/19091995/

23. Chaguza C, Yang M, Cornick JE, du Plessis M, Gladstone RA, Ba KA, et al. Bacterial genome-wide association study of hyper-virulent pneumococcal serotype 1 identifies genetic variation associated with neurotropism. Commun Biol [Internet]. 2020 Oct 8 [cited 2024 Nov 4];3(1). Available from: https://pubmed.ncbi.nlm.nih.gov/33033372/

24. Plessis M du, Allam M, Tempia S, Wolter N, Gouveia L de, Mollendorf C von, et al. Phylogenetic Analysis of Invasive Serotype 1 Pneumococcus in South Africa, 1989 to 2013. J Clin Microbiol. 2016 Apr 25;54(5):1326.

25. Yaro S, Lourd M, Traoré Y, Njanpop-Lafourcade BM, Sawadogo A, Sangare L, et al. Epidemiological and molecular characteristics of a highly lethal pneumococcal meningitis epidemic in Burkina Faso. Clin Infect Dis Off Publ Infect Dis Soc Am. 2006 Sep 15;43(6):693–700.

26. Antonio M, Dada-Adegbola H, Biney E, Awine T, O'Callaghan J, Pfluger V, et al. Molecular epidemiology of pneumococci obtained from Gambian children aged 2-29 months with invasive pneumococcal disease during a trial of a 9-valent pneumococcal conjugate vaccine. BMC Infect Dis. 2008 Jun 11;8:81.

27. Kwambana-Adams BA, Asiedu-Bekoe F, Sarkodie B, Afreh OK, Kuma GK, Owusu-Okyere G, et al. An outbreak of pneumococcal meningitis among older children (≥5 years) and adults after the implementation of an infant vaccination programme with the 13-valent pneumococcal conjugate vaccine in Ghana. BMC Infect Dis. 2016 Oct 18;16(1):575.

28. Zemlicková H, Crisóstomo MI, Brandileone MC, Camou T, Castañeda E, Corso A, et al. Serotypes and clonal types of penicillin-susceptible streptococcus pneumoniae causing invasive disease in children in five Latin American countries. Microb Drug Resist Larchmt N. 2005;11(3):195–204.

29. Almeida S, de Lencastre H, Sá-Leão R. Epidemiology and population structure of serotypes 1, 5 and 7f carried by children in Portugal from 1996-2010 before introduction of the 10-valent and 13-valent pneumococcal conjugate vaccines. PloS One [Internet]. 2013 Sep 18 [cited 2024 Nov 4];8(9). Available from: https://pubmed.ncbi.nlm.nih.gov/24058686/

30. Horácio AN, Silva-Costa C, Diamantino-Miranda J, Lopes JP, Ramirez M, Melo-Cristino J, et al. Population Structure of Streptococcus pneumoniae Causing Invasive Disease in Adults in Portugal before PCV13 Availability for Adults: 2008-2011. PloS One. 2016;11(5):e0153602.

31. Serrano I, Melo-Cristino J, Carriço JA, Ramirez M. Characterization of the genetic lineages responsible for pneumococcal invasive disease in Portugal. J Clin Microbiol. 2005 Apr;43(4):1706–15.

32. Clarke SC, Jefferies JMC, Smith AJ, McMenamin J, Mitchell TJ, Edwards GFS. Pneumococci causing invasive disease in children prior to the introduction of pneumococcal conjugate vaccine in Scotland. J Med Microbiol. 2006 Aug;55(Pt 8):1079–84.

33. Cooke B, Smith A, Diggle M, Lamb K, Robertson C, Inverarity D, et al. Antibiotic resistance in invasive Streptococcus pneumoniae isolates identified in Scotland between 1999 and 2007. J Med Microbiol. 2010 Oct;59(Pt 10):1212–8.

34. Foster D, Knox K, Walker AS, Griffiths DT, Moore H, Haworth E, et al. Invasive pneumococcal disease: epidemiology in children and adults prior to implementation of the conjugate vaccine in the Oxfordshire region, England. J Med Microbiol. 2008 Apr;57(Pt 4):480–7.

35. Grau I, Ardanuy C, Calatayud L, Rolo D, Domenech A, Liñares J, et al. Invasive pneumococcal disease in healthy adults: increase of empyema associated with the clonal-type Sweden(1)-ST306. PloS One. 2012;7(8):e42595.

36. Muñoz-Almagro C, Jordan I, Gene A, Latorre C, Garcia-Garcia JJ, Pallares R. Emergence of invasive pneumococcal disease caused by nonvaccine serotypes in the era of 7-valent conjugate vaccine. Clin Infect Dis Off Publ Infect Dis Soc Am. 2008 Jan 15;46(2):174–82.

37. Byington C, Hulten KG, Ampofo K, Shen X, At P, Aj B, et al. Molecular epidemiology of pediatric pneumococcal empyema from 2001 to 2007 in Utah. J Clin Microbiol [Internet]. 2010 Feb [cited 2024 Nov 4];48(2). Available from: https://pubmed.ncbi.nlm.nih.gov/20018815/

38. Lai JY, Cook H, Yip TW, Berthelsen J, Gourley S, Krause V, et al. Surveillance of pneumococcal serotype 1 carriage during an outbreak of serotype 1 invasive pneumococcal disease in central Australia 2010-2012. BMC Infect Dis. 2013 Sep 3;13:409.

39. Hanachi M, Kiran AM, Cornick JE, Harigua-Souiai E, Everett D, Benkahla A, et al. Genomic Characteristics of Invasive Streptococcus pneumoniae Serotype 1 in New Caledonia Prior to the Introduction of PCV13. Bioinforma Biol Insights [Internet]. 2020 Sep 29 [cited 2024 Nov 4];14. Available from: https://pubmed.ncbi.nlm.nih.gov/33088176/

40. Jefferies JM, Smith AJ, Edwards GFS, McMenamin J, Mitchell TJ, Clarke SC. Temporal analysis of invasive pneumococcal clones from Scotland illustrates fluctuations in diversity of serotype and genotype in the absence of pneumococcal conjugate vaccine. J Clin Microbiol. 2010 Jan;48(1):87–96.

41. Zähner D, Gudlavalleti A, Stephens DS. Increase in Pilus Islet 2–encoded Pili among Streptococcus pneumoniae Isolates, Atlanta, Georgia, USA. Emerg Infect Dis. 2010 Jun;16(6):955.

42. Horácio AN, Diamantino-Miranda J, Aguiar SI, Ramirez M, Melo-Cristino J. Serotype changes in adult invasive pneumococcal infections in Portugal did not reduce the high fraction of potentially vaccine preventable infections. Vaccine. 2012 Jan 5;30(2):218–24.

43. Gonzalez BE, Hulten KG, Kaplan SL, Mason EO, US Pediatric Multicenter Pneumococcal Surveillance Study Group. Clonality of Streptococcus pneumoniae serotype 1 isolates from pediatric patients in the United States. J Clin Microbiol. 2004 Jun;42(6):2810–2.

44. Zhou H, Guo J, Qin T, Ren H, Xu Y, Wang C, et al. Serotype and MLST-based inference of population structure of clinical Streptococcus pneumoniae from invasive and noninvasive pneumococcal disease. Infect Genet Evol J Mol Epidemiol Evol Genet Infect Dis. 2017 Nov;55:104–11.

45. Donkor ES, Adegbola RA, Wren BW, Antonio M. Population biology of Streptococcus pneumoniae in West Africa: multilocus sequence typing of serotypes that exhibit different predisposition to invasive disease and carriage. PloS One. 2013;8(1):e53925.
